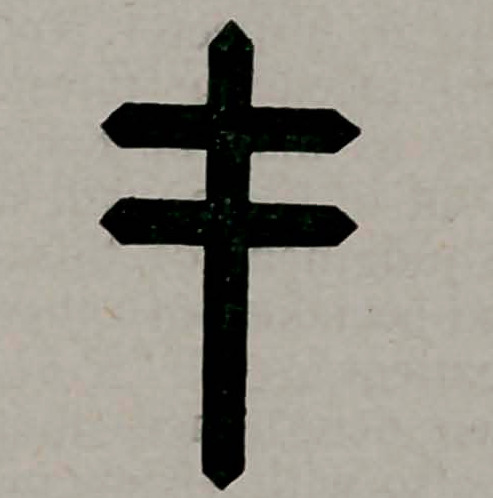# Topics of Public Interest

**Published:** 1910-10

**Authors:** 


### TOPICS OF PUBLIC INTEREST

### General Leonard Wood at the Head of the Army.

**New Chief of Staff says success comes easily—Some Characteristic Anecdotes that are told about the "Fighting Doctor."**

[*The Tribune, July 24, 1910.*]

"SUCCESS is so easy that it is a crime to fail."

Such was the comment of General Leonard Wood when asked by what secret method of wooing fame he had won a career equalled by few American soldiers. For the "Fighting Doctor" and Rough Rider colonel is just taking up his duties as chief of staff and head of the United States army.

Success hasn't seemed to be much of a trick for square-shouldered, modest Dr. Wood. The old folks around Cape Cod, where he grew up, agree that "Len Wood never made much noise, but he just naturally couldn't help being a leader." His father was a physician, and the boy studied medicine and practised it. Even when he contracted the army fever, he joined the service as a doctor, but—well, the Indians made so much trouble that Dr. Wood "just couldn't help" dropping his medicine case and pitching into the fight. Then he simply had to take charge of things, and here he is.

Success with General Wood isn't measured by the annual returns in greenbacks, either. A few years ago the Washington Traction and Electric Company, whose directors had been watching the soldier-doctor's executive feats in Cuba, offered him the company's presidency.

"I will not leave the military service of the United States so long as my services are wanted—not for $30,000 a year, or twice that amount," he said when some one asked what he was going to do about it. "Money is not the only thing in the world," he added.

His first advance in military favor was won with his fists. Curiously enough, the man he knocked out was the commanding officer of his department, later Major General Miles. Young Wood had just entered the army. He was then, as now, strong as a mogul engine and tough as tempered steel, with powerful shoulders and arms, short, thick neck and sturdy legs. He was an all around athlete and particularly expert as a boxer. Fistic bouts were a favorite pastime at headquarters. Miles was proud of his own prowess. The shy young surgeon hung back, however, till the general one night gave him a special invitation to put on the gloves, assuring him he would take into consideration his opponent's youth, and the like. That made Dr. Wood mad through and through. As a result, General Miles was thoroughly thrashed, and from that time forth was the warm friend of the young doctor, who was destined to stand one day in his shoes as head of the army.

He was still as unspoiled and modest as ever when he was picked as the man to govern Cuba. When a friend rushed to congratulate him on the appointment he waved him back with the laconic remark, "Wait a year."

One of the first things the Cubans noticed about the new Governor, aside from his modesty, was his eminent fairness. To begin with, he actually paid for his newspapers, a thing previously unheard of in Cuban official circles. Commenting on this, the "Diario de la Marina" said:

"It is the first time in the history of the island when a Governor General has either paid or offered to pay a newspaper subscription. Hitherto it has been the custom of governors general to order not one copy of each issue, as General Wood has done, but several copies. This departure from the precedents gives General Wood a unique distinction. Native officials of minor rank holding office in the city might with propriety do likewise."

Nor was there any sentimentality or weakness mingled with his love for the "square deal." Reports came to him that American soldiers were in the habit of eating at Cuban restaurants and refusing to pay. Other places of business suffered likewise. One day a volunteer soldier was caught raiding a jewelry store with four fellow soldiers, who escaped.

"Do you know what the penalty of this offence is if you are proved guilty?" demanded General Wood sternly.

The fellow didn't, nor did he greatly care. He took it all as a joke, expecting his offence to be winked at after a perfunctory lecture.

"Well, you will be hanged," returned the general. "Now, tell me who were with you?"

The man wilted at once, and a half hour later all five were in the guardhouse awaiting trial.

He soon came to be regarded as a Daniel come to judgment. The limit was reached, however, when he was called upon to settle a squabble between a landlord and tenant as to the amount of rent due. He announced that the civil authorities must settle that, but added that in case the matter was decided against the tenant he could be counted on to throw the man and his goods into the street.

He found the price of meat too high in Santiago. The butchers were summoned before him without delay. Through an interpreter he asked:

"How much do you charge for your meat?"

"Ninety cents a pound, señor."

"What does it cost you?"

There was hesitation and a shuffling of feet; then one of the men said, in a whining voice:

"Meat is very, very dear, your excellency."

"How much a pound?"

"It costs us very much, and"——

"How much a pound?"

"Fifteen cents, your excellency, but we have lost much money during the war and"——

"So have your customers. Now meat will be sold at 25 cents a pound, and not one cent more. Do you understand?"

They did.

That typical Cuban institution, the agitating editor, began making him trouble as soon as he struck the island. He sent for the most violent of the editors.

"You may say anything you please against me personally," he said in his quiet way, "but the moment you attack the government I shall put you in Morro Castle and keep you there."

Another one of these editors had suggested "going to the hills," which in Cuba means rebellion. Wood sent for him, too, and told him that the sooner he went to the hills the better it would be for his own safety, and he said it so seriously that the next day the editor did go to the hills.

General Wood sitting in judgment in a Cuban jail was a sight to remember. He never forgot a face, never overlooked a loophole in the evidence, never allowed himself to be hoodwinked. A man was brought before him charged with stealing oats.

"Ever arrested before?" asked the general.

There was a search of records and the jailer shook his head.

"Look again," exclaimed General Wood, sternly. "I remember him. He was here a month ago. Been stealing on the wharf. Hold him for trial. Next.

Another prisoner was an American soldier arrested for drunkenness and shooting a revolver in Calle Santo Tomas at midnight.

"What have you to say for yourself?" demanded General Wood. "Are you guilty?"

The man shook his head and grinned insolently.

In an instant the general was on his feet. With one stride he was beside the prisoner.

"You will find this is no laughing matter," he thundered. "Stand erect. Put your heels together. Now answer me. Did you fire a revolver in the street, as accused?"

"Yes, sir," the fellow stammered.

"Ten days," ordered the general.

"Thank you, sir," muttered the fellow under his breath as he turned away. But the general heard it.

"Make that ten days in the sweat box," came the curt command.

The man passed out into the darkness, and a second later a defiant laugh was heard in his direction.

"Make it bread and water also," added the general. "If he causes any trouble put him in irons. Next."

On another memorable occasion General Wood was writing in his office in the palace one evening. A solitary sentry, armed with a rifle, stood at the outer door. Suddenly there burst across the plaza a mob of five hundred Cubans. A shower of stones, bricks and other missiles were poured through the windows of the Spanish Club. A hatless messenger came rushing in shouting "Where's the general? Quick! The Cubans are trying to take the town!"

General Wood was leisurely folding up his papers. "I know it," he calmly remarked to the breathless messenger. "I have heard the row. We will go over and stop it."

Picking up his riding whip, usually his only weapon, accompanied by the lone American soldier, he strolled across to the scene of trouble. Reaching the door of the club, the general and his single guard turned.

"Shove them back, sentry," he commanded.

Around swung the rifle, and a way was soon cleared in front of the door.

"Now shoot the first man who places his foot on that step," he said and strolled back to his writing. Within an hour the mob had dispersed.

Thus, calmly, fearlessly, vigorously the "Fighting Doctor" has wrought his career. Wherever he has gone reforms have followed. Now that he has the entire military force of the country at his disposal his friends predict that "things will look up all along the line."

General Wood, like all great fighters, is a man of peace. Yet he considers "militarism," that terrible word so often conjured with, a mere bugaboo and denies that the huge standing armies and universal military service of Europe are a misfortune industrially. On the contrary, he believes such service of the highest educational value in subsequent civil pursuits and such preparedness for war the surest guarantee of peace.

Not that he would apply that system in this country. He freely admits that at the present time public sentiment here would not tolerate a larger standing army or any measure of compulsory military service. However, he has in mind definite policies for increasing the efficiency of the standing army and the size and effectiveness of the national guard. General Wood therefore believes in:

Doubling the attendance at West Point so that there shall be twice as many trained officers available. The general, though not a West Point man, says he would be twice as useful if he were.

Strengthening the service schools.

A more definite schooling system for the national guard, beginning with elementary lessons the first year, field maneuvres the second, and closing with sham battles in the third. Every inducement should be offered to get men into the national guard. Employers of labor should remove all restrictions that today often keep men out.

Shortening the term of enlistment in the regular army so that the force shall be made up of younger men and more trained soldiers turned back to civil life to form the nucleus of national guard bodies.

Government ownership of camp sites.

Fairer treatment of army convicts and reinstatement of men dismissed for minor offences.

A well regulated canteen as a conserver of army morals.

The development of an efficient aeronautical service.

Along all these lines the silent, active doctor-soldier is saying little, but it is confidently believed he is thinking a lot.

### United States Bureau of the Census

**Advance Vital Statistics—Infantile Paralysis and Pellagra—Number of Deaths from Each in 1909 in the Census Bureau's Registration Area.**

THERE were 569 deaths from acute anterior poliomyelitis, or infantile paralysis, 116 from pellagra, 55 from rabies, or hydrophobia, and 9 deaths from leprosy in 1909, in the death registration area of continental United States, which comprises over 55 per cent. of the total population, according to the Census Bureau's forthcoming bulletin on mortality statistics for 1909 submitted to Census Director Durand by Dr. Cressy L. Wilbur, chief statistician for vital statistics.

It is reported that, of the 569 deaths from infantile paralysis, 522 were of white and only 17 of colored persons. There was a somewhat greater incidence of disease among males and an increased mortality in August, September, and October.

The bulletin states that no statistical segregation of infantile paralysis as a cause of death has been made heretofore, but the increasing importance of the disease and its wide prevalence throughout the country in the form of local epidemics render a statement of the mortality important. Like meningitis, which it somewhat resembles, it is difficult to obtain an exact separation of the deaths from the specific infectious disease, acute anterior poliomyelitis, from other infections of similar nature. Acute anterior poliomyelitis is described by the bulletin as an acute infectious disease chiefly affecting children in the first five years of life, and while not infrequently fatal is of even more serious consequence as the cause of more or less permanent paralysis and atrophy of muscles. Numerous outbreaks have occurred in this country, the most important of which were those in Vermont, in 1894, and in New York and Connecticut, in 1907. The 569 deaths compiled for the registration area for 1909 were widely distributed, and indicate endemic or epidemic prevalence in many parts of the country. It should be remember, the bulletin points out, that the census data relate only to registration sources, and that for the nonregistration states the deaths are only those returned from the registration cities contained therein. The deaths from acute anterior poliomyelitis in the registration states numbered as follows: California, 12 (1 in San Francisco); Colorado, 6 (1 in Denver); Connecticut, 6 (1 in New Haven); District of Columbia (city of Washington), 1; Indiana, 14; Maine, 6; Maryland, 4 (1 in Baltimore); Massachusetts, 62 (21 in Boston and 1 in Worcester); Michigan, 16 (2 in Detroit); New Hampshire, 11; New Jersey, 24 (2 in Jersey City, 6 in Newark); New York, 115 (2 in Buffalo, 64 in Greater New York, 2 in Rochester, 1 in Syracuse); Ohio, 16 (1 in Cincinnati, 2 in Cleveland; Pennsylvania, 76 (8 in Philadelphia, 8 in Pittsburg, 1 in Scranton); Rhode Island, 4 (3 in Providence); South Dakota, 6; Vermont, 2; Washington, 5; and Wisconsin, 51 (1 in Milwaukee).

The disease does not seem particularly to affect the large cities of 100,000 and over population in 1900, as given in the preceding list.

For the nonregistration states there were, in the registration cities only, deaths numbering as follows: Alabama, 2; Illinois, 19 (17 in Chicago); Kentucky, 2 (1 in Louisville); Louisiana, 1 (New Orleans); Minnesota, 82 (21 in Minneapolis, 53 in St. Paul); Missouri, 5 (1 in Kansas City, 4 in St. Louis); Nebraska, 8 (Omaha); North Carolina, 1; Oregon, 2 (Portland); South Carolina, 1 (Charleston); Tennessee, 1; Texas, 2; Utah, 3, and Virginia, 3.

The duration of illness prior to death was reported only in 292 of the 569 cases. In 19 of these it was 1 year or more, 1 each being reported as of 10, 14, 16, 18, and 20 years' duration. These may represent the results of old attacks or perhaps may include deaths from other forms of poliomyelitis. There were 40 cases of duration of illness in excess of 1 month but less than a year, 21 of which were under 2 months. The great majority of the fatal cases returned were of very brief duration, 253, or 87 per cent., being of less than 1 month. Of these there were 20 stated to be of 1 day, 22 of 2 days, 30 of 3 days, 31 of 4 days, 28 of 5 days, 18 of 6 days, 24 of 7 days, 10 of 8 days, 5 of 9 days, 22 of 10 days, only 1 of 11 days. The tendency to report in round numbers or to give even weeks somewhat vitiates the exact statements; the average duration of all the fatal cases with duration of under 1 month is 7.2 days.

The bulletin states that among the rarer diseases included in the epidemic group may be found some whose occasional occurrence awakens more interest and popular fear than many hundred times as many deaths from more accustomed causes. Among these there were, during the year 1909, 3 deaths from typhus (typhus fever), 79 deaths from smallpox, 2 deaths from plague, and 9 deaths from leprosy. No deaths occurred from Asiatic cholera or from yellow fever.

In the second subdivision of the class of general diseases there were compiled 8 deaths from glanders, 14 from anthrax (malignant pustule), 55 from rabies (hydrophobia), 38 from actinomycosis, trichinosis, and the like, 116 from pellagra, 86 from lead poisoning, and 5 from other occupational poisoning.

Pellagra is a new disease in the mortality statistics the bulletin states. Only 23 deaths were returned from this cause for 1908, and no deaths for any previous year except 1 for 1904. Such deaths undoubtedly occurred, but were not recognised and were consequently returned as due to other causes or as of unknown cause.

As the registration area includes only a small portion of the country in which pellagra is most prevalent, it would seem that many hundred and perhaps thousands of deaths from this disease must occur each year in the United States. How many can never be known until systems of complete registration of deaths are more generally adopted.

Washington, D. C., September 24, 1910.

---

### How the Double Red Cross Originated

### International Tuberculosis Emblem Adopted in 1902

ALTHOUGH the double red cross has been used in America for more than four years as the international emblem of the crusade against tuberculosis, few people have known how it originated until announcement of the history of the symbol was made public today by the National Association for the Study and Prevention of Tuberculosis.

It has been ascertained that the double red cross was first suggested as the symbol of the International Antituberculosis Association in Berlin in October, 1902. The proposer was Dr.

G. Sersiron of Paris who is now Associate Secretary of L'Association Centrale Française Contre la Tuberculose. Dr. Sersiron's proposal was adopted at the Berlin meeting and a movement was at once started to secure official recognition and protection for the double cross from European governments.

The double red cross is similar in shape to a cross used frequently in the Greek Catholic Churches, and also to the Lorraine Cross of France. The National Association for the Study and Prevention of Tuberculosis in the United States has adopted the proportions of nine for the length of the cross to five for the width of the arms, with a space one-ninth of the length between the arms.

In 1902, when the double red cross was adopted, there were not more than a half-dozen associations for the prevention of tuberculosis organised on a wide basis. Today under the banner of the antituberculosis crusade, associations have been formed in almost every civilised country in the world. Even China is beginning to take action along this line, while in Turkey, India, Japan, the Philippines, South Africa, Australia, Iceland, and in all of the European countries active societies are at work. In the United States, from four independent associations in 1902, the double red cross now enlists a carefully organised national movement under which are affiliated more than thirty state bodies and 420 local societies. If to these agencies are added the local, state, and national governments enrolled in antituberculosis work, the double red cross becomes the symbol of the greatest organised campaign for the prevention of disease that the world has ever known.

## Figures and Tables

**Figure f1:**